# Primary cilia mediate Klf2-dependant Notch activation in regenerating heart

**DOI:** 10.1007/s13238-020-00695-w

**Published:** 2020-04-05

**Authors:** Xueyu Li, Qiang Lu, Yuanyuan Peng, Fang Geng, Xuelian Shao, Huili Zhou, Ying Cao, Ruilin Zhang

**Affiliations:** 1 School of Life Sciences, Fudan University, Shanghai, 200433 China; 2 Shanghai Medical College, Fudan University, Shanghai, China; 3 Department of Molecular and Cell Biology, School of Life Sciences and Technology, Tongji University, Shanghai, 200331 China; 4 School of Basic Medical Sciences, Wuhan University, Wuhan, 430072 China; 5 State Key Laboratory of Genetic Engineering, Fudan University, Shanghai, 200433 China

**Keywords:** heart regeneration, hemodynamics, *klf2*, Notch signaling, primary cilia

## Abstract

Unlike adult mammalian heart, zebrafish heart has a remarkable capacity to regenerate after injury. Previous study has shown Notch signaling activation in the endocardium is essential for regeneration of the myocardium and this activation is mediated by hemodynamic alteration after injury, however, the molecular mechanism has not been fully explored. In this study we demonstrated that blood flow change could be perceived and transmitted in a primary cilia dependent manner to control the hemodynamic responsive *klf2* gene expression and subsequent activation of Notch signaling in the endocardium. First we showed that both homologues of human gene *KLF2* in zebrafish, *klf2a* and *klf2b*, could respond to hemodynamic alteration and both were required for Notch signaling activation and heart regeneration. Further experiments indicated that the upregulation of *klf2* gene expression was mediated by endocardial primary cilia. Overall, our findings reveal a novel aspect of mechanical shear stress signal in activating Notch pathway and regulating cardiac regeneration.

**Electronic supplementary material:**

The online version of this article (10.1007/s13238-020-00695-w) contains supplementary material, which is available to authorized users.

## Introduction

Adult mammalian heart cannot replenish lost cardiomyocytes (CMs) resulted from myocardial infarction (MI) which could eventually lead to heart failure (Thygesen et al., 2007). By contrast, zebrafish have a strong capacity to regenerate injured heart through dedifferentiation and proliferation of pre-existing CMs (Poss et al., 2002; Jopling et al., 2010; Kikuchi et al., 2010). Notch signal pathway plays a pivotal role in multiple tissue repair processes including zebrafish heart regeneration (Raya et al., 2003). After genetic ablation or surgical resection, Notch signaling is activated in the endocardium of injured heart and subsequently leads to heart regeneration, whereas blockage of such activation impedes this regenerative process (Zhang et al., 2013; Zhao et al., 2014). Despite extensive studies have been carried out on the function of Notch signaling in heart regeneration, the molecular mechanism how it is activated has not been fully elucidated yet (Felician et al., 2014; Nemir et al., 2014; Munch et al., 2017; Zhao et al., 2019).

Hemodynamic force is crucial for the formation of heart valves and cardiac trabeculae, the development and maintenance of hematopoietic stem cells, and the cause of various cardiovascular diseases, frequently acting through Notch signal pathway (Vermot et al., 2009; Nixon et al., 2010; Samsa et al., 2015; Liu et al., 2019). Many factors have been reported to perceive and transmit mechanical shear stress signal, including primary cilia, mechanical-sensitive ion channels, cell adhesion molecules, protein receptor family, transcription factors, etc (Baratchi et al., 2017). Galvez-Santisteban et al. recently reported alteration of intracardiac blood flow and hemodynamic shear-stress after ventricle ablation, which was essential for endocardial Notch activation (Gálvez-Santisteban et al., 2019). This activation was mediated by *klf2a,* zebrafish homologue of the human Krüppel-like factor 2 gene (*KLF2*) which encodes a well-known hemodynamic responsive transcription factor playing a crucial regulatory role in vascular and cardiac morphogenesis *in vivo* (Lee et al., 2006; Goddard et al., 2017). However, the function of the other homologue *klf2b* or the identity of factors which perceive and transmit mechanical shear stress signal in this process remains to be elucidated.

This study is aimed to explore the underlying molecular mechanisms of sensation and transmission of mechanical hemodynamic signal to Notch activation during cardiac regeneration. First we demonstrated that both *klf2a* and *klf2b* could respond to hemodynamic alteration, whereas Notch signaling activation and heart regeneration were impeded in *klf2* single or double mutants. Further experiments indicated that endocardial primary cilia mediated the upregulation of *klf2* gene expression and the subsequent activation of Notch signal pathway. Overall, our findings reveal a novel aspect of mechanical shear stress signal in stimulating Notch activation and regulating cardiac regeneration.

## Results

### Reduced blood flow attenuates Notch signaling activation and inhibits ventricle regeneration

To visualize the transient activation of Notch signaling in the endocardium of regenerating hearts, we used a reporter line *Tg(tp1:d2GFP)* expressing destabilized GFP in Notch-activated cells, to cross with a ventricle ablation line *Tg(vmhc:mCherry-NTR)* (Clark et al., 2012; Zhang et al., 2013). DMSO-treated control group displayed strong Notch signals in the atrioventricular canal (AVC) and weak signals in the outflow tract (OFT) (Supplementary figure 1A–C). After ventricle injured with metronidazole (MTZ) treatment at 3 days post fertilization (dpf), Notch signaling was activated in the ablated heart mainly in the AVC and the ventricle, also extending from the AVC to the atrium in various degrees (Supplementary figure 1D–F).

Galvez-Santisteban et al. recently displayed increased level of oscillatory fluctuations of anterograde and retrograde intracardiac flow in injured heart by particle image velocimetry (PIV) analyses (Gálvez-Santisteban et al., 2019) and proved this high oscillatory flow was required for Notch activation. To temporarily inhibit blood flow after ventricle ablation, we utilized Tricaine and 2,3-Butanedione monoxime (BDM), two muscle relaxants affecting electrical function and myosin contraction respectively. Larvae were treated with Tricaine or BDM at 15–24 hpt to achieve most significant effect in ablated group yet with no adverse phenotype observed in non-ablated control group. Notch signal activation was totally blocked in the 24 hpt ablated heart right after Tricaine or BDM treatment (Supplementary figure 1G–J). Whole-mount *in situ* hybridizations (WISH) also showed significantly attenuated *notch1b* mRNA upregulation in ablated hearts at 24 hpt with Tricaine or BDM treatment (Supplementary figure 1K–P).

This brief inhibition of blood flow and Notch signaling was sufficient to disturb heart regeneration. Compared to ablated larvae without treatment, the ventricular fluorescence and heart morphology of ablated larvae treated with Tricaine or BDM could not restore at 4 days post treatment (dpt) (Supplementary figure 2). Quantification of regeneration rate at 4 dpt showed that the percentage of recovered larvae after reducing blood flow dropped to 20% in Tricaine treated ablated group (N = 336) and 21% in BDM treated ablated group (N = 126), significantly lower than the 83% recovery rate in wildtype ablated group (N = 249) (Supplementary figure 1Q). Overall, our results confirmed that hemodynamic force was essential for the activation of Notch signaling and ventricle regeneration in agreement with recent finding by Galvez-Santisteban et al. (Gálvez-Santisteban et al., 2019).

### Reduced blood flow changes *klf2a* and *klf2b* gene expression during ventricle regeneration

**Figure 1:**
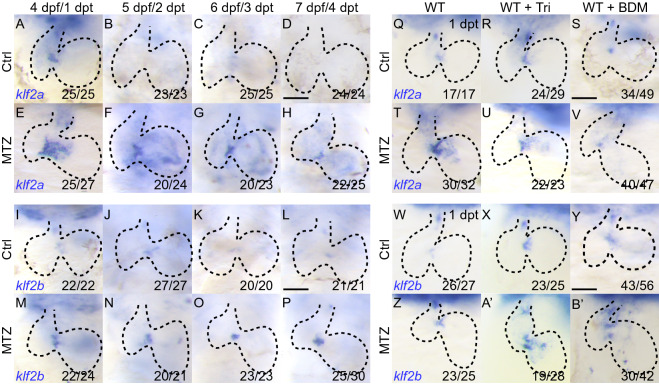
**Reduced blood flow changes*klf2a*and*klf2b*expression during ventricle regeneration**. (A–H) Whole-mount *in situ* hybridizations showing *klf2a* upregulation in ablated hearts (E–H) compared to control hearts (A–D) at 4–7 dpf/1–4 dpt. (I–P) Whole-mount *in situ* hybridizations showing *klf2b* upregulation in ablated hearts (M-P) compared to control hearts (I–L) at 4–7 dpf/1–4 dpt. (Q–V) Whole-mount *in situ* hybridizations indicated *klf2a* upregulation after ablation (Q, T) was blocked in Tricaine treated hearts (R, U) and BDM treated hearts (S, V) at 1 dpt. (W–B’) Whole-mount *in situ* hybridizations indicated *klf2b* upregulation after ablation (W, Z) was slightly enhanced in Tricaine treated hearts (X, A’) and BDM treated hearts (Y, B’) at 1 dpt. Scale bars, 50 µm. dpt, days post treatment. Dashed lines outline the hearts. Numbers indicate the ratio of representative staining observed

KLF2 is well-known for its responsiveness to hemodynamic alteration during mammalian development and various disease progressions (Dekker et al., 2002). The zebrafish homologue *klf2a* has been proved to play a conserved role in similar settings (Steed et al., 2016), however, the function of the other homologue *klf2b* has not been fully characterized (Oates et al., 2001). We first used WISH to compare the expression profiles of both homologues during ventricle regeneration. Both *klf2a* and *klf2b* genes were expressed weakly in the AVC and OFT of control larvae hearts at 4 dpf, then *klf2a* expression decreased from 5 to 7 dpf whereas *klf2b* expression remained constant (Fig. 1A–D, I–L). After cardiac injury at 1 dpt, *klf2a* expression was significantly upregulated in the AVC where the level of oscillatory flow increased the most. From 2 to 4 dpt, *klf2a* expression was gradually weakened in the AVC, but the range spread to the ventricle and atrium (Fig. 1E–H). By contrast, the expression of *klf2b* in ablated hearts was slightly upregulated in the AVC at 1 dpt, which maintained a constant level and did not extend to either chamber at later stages (Fig. 1M–P). We then examined how *klf2a* and *klf2b* genes responded to reduced blood flow. The upregulation of *klf2a* mRNA in the AVC of ablated hearts at 1 dpt was attenuated upon Tricaine or BDM treatment (Fig. 1Q–V). Interestingly, *klf2b* expression in some of the Tricaine or BDM treated control and ablated hearts was slightly enhanced and expanded from the AVC (Fig. 1W–B’). These results suggested that *klf2a* and *klf2b* genes responded differently to blood flow alteration and might play different roles during cardiac regeneration.

### *klf2* mutants result in compensation of homologue expression

**Figure 2:**
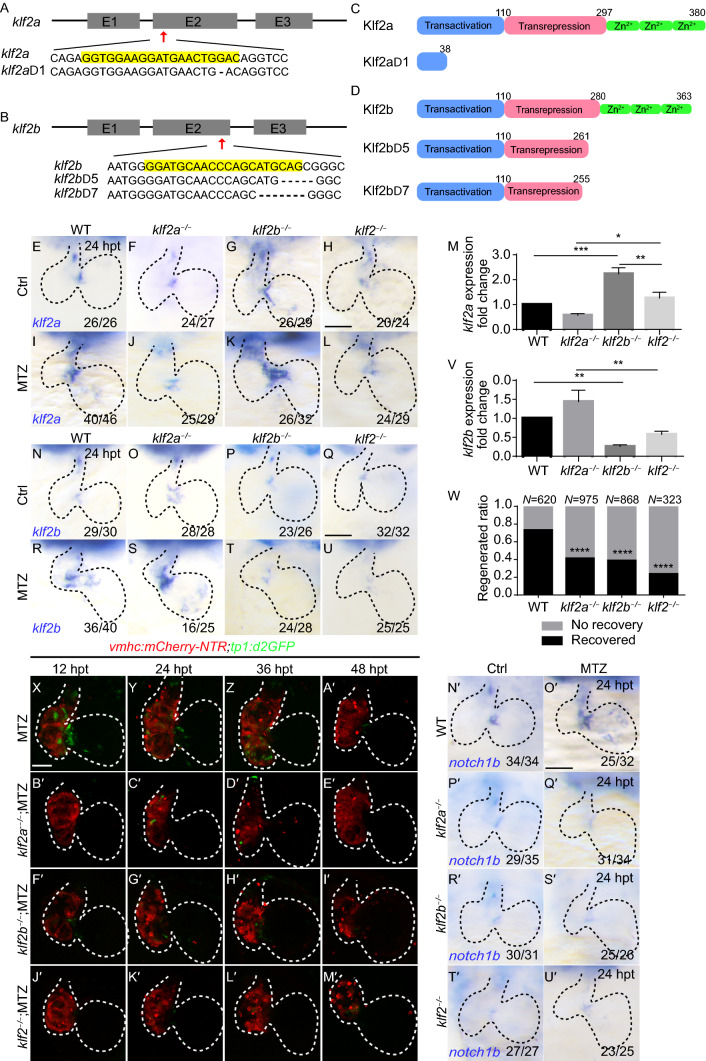
***klf2*mutants result in compensation of homologue expression, blunted Notch signaling activation and reduced ventricle regeneration**. (A and B) Schematic diagrams of the zebrafish *klf2a* and *klf2b* loci with the sgRNA target site sequence (red arrowheads). (C, D) Functional domain diagrams of wildtype Klf2a or Klf2b and predicted truncated protein in corresponding mutants. (E–L) Whole-mount *in situ* hybridizations showing *klf2a* expression pattern in the control and ablated hearts of wildtype, *klf2a*^−/−^ mutants, *klf2b*^−/−^ mutants and *klf2*^−/−^ double mutants at 24 hpt. (M) Quantification of the fold change of *klf2a* expression in wildtype and *klf2* mutant larvae at 4 dpf by real time PCR. 4 independent experiments. Mean + s.e.m. ANOVA analysis, *P < 0.05, **P < 0.01, ***P < 0.001. (N–U) Whole-mount *in situ* hybridizations showing *klf2b* expression pattern in the control and ablated hearts of wildtype, *klf2a*^−/−^ mutants, *klf2b*^−/−^ mutants and *klf2*^−/−^ double mutants at 24 hpt. (V) Quantification of the fold change of *klf2b* expression in wildtype and *klf2* mutant larvae at 4 dpf by real time PCR. 4 independent experiments. Mean + s.e.m. ANOVA analysis, **P < 0.01. (W) Quantification of the heart recovery rate (black bars) in ablated wildtype and *klf2* mutants at 4 dpt. The number of larvae analyzed for each condition is indicated. Binomial test (versus WT), ****P < 0.0001. (X–M’) Confocal stack projections of ablated *Tg*(*vmhc*:*mCherry-NTR*; *tp1*:*d2GFP*) hearts showing Notch signaling pattern in wildtype (X–A’), *klf2a*^−/−^ mutants (B’–E’), *klf2b*^−/−^ mutants (F’–I’) and *klf2*^−/−^ double mutants (J’–M’) at 12, 24, 36, 48 hpt. (N’–U’) Whole-mount *in situ* hybridizations indicated *notch1b* upregulation as in ablated wildtype hearts (N’, O’) was blocked in *klf2a*^−/−^ mutant hearts (P’, Q’), *klf2b*^−/−^ mutant hearts (R’, S’) and *klf2*^−/−^ double mutant hearts (T’, U’) at 24 hpt. Scale bars, 50 µm. hpt, hours post treatment. Dashed lines outline the hearts. Numbers indicate the ratio of representative staining observed

To further investigate the role of *klf2a* and *klf2b* in the regulation of cardiac regeneration, we generated mutants using CRISPR/Cas9 genome editing technique. We first obtained *klf2a*^−/−^ frameshift mutants bearing 1 bp deletion which resulted in premature translation termination. The predicted truncated protein consisted of 38 amino acids compared to the full length of 380 amino acids in wildtype Klf2a (Fig. 2A, C). We next obtained *klf2b*^−/−^ mutants bearing 5 bp deletion which produced truncated proteins of 261 amino acids. The *klf2a*^−/−^/*klf2b*^−/−^ double mutants (referred as *klf2*^−/−^ double mutants later on) with 1-bp-deletion in *klf2a* and 7-bp-deletion in *klf2b* produced truncated proteins of similar size, missing the conserved zinc finger domains (Fig. 2B, D). During the first week of development most larvae of *klf2a*^−/−^ mutants, *klf2b*^−/−^ mutants and *klf2*^−/−^ double mutants did not show gross morphological defects. We found a small percentage of larvae with cardiac edema in *klf2* single and double mutants but no obvious CM extrusion was observed in double mutants, which was different from a previous report (Rasouli et al., 2018). Although *klf2a*^−/−^ and *klf2b*^−/−^ single mutants could grow to adulthood normally, most of *klf2*^−/−^ double mutant fish would die during the second week of development due to unidentified reasons, and only a few could reach sexual maturity but still were prone to death.

To assess the knockout efficiency we performed real time PCR using cDNA from whole mutant larvae at 4 dpf (Fig. 2M, V, 4 independent experiments). *klf2a* mRNA level was reduced to 0.57 ± 0.06 fold of wildtype in *klf2a*^−/−^ larvae whereas *klf2b* mRNA level had a larger reduction to 0.26 ± 0.05 fold of wildtype in *klf2b*^−/−^ larvae. Interestingly, *klf2a* mRNA level significantly increased to 2.23 ± 0.25 fold in *klf2b*^−/−^ larvae while *klf2b* mRNA level showed a similar trend in *klf2a*^−/−^ larvae with 1.44 ± 0.30 fold compared to wildtype, suggesting a possible compensation of *klf2* homologue expression in single mutants. The expression level of *klf2a* or *klf2b* in *klf2*^−/−^ double mutants was between the levels in two single mutants, maybe due to the average of reduction and compensation effects. WISH of *klf2a* and *klf2b* mRNA in the hearts of wildtype, *klf2a*^−/−^ mutants, *klf2b*^−/−^ mutants and *klf2*^−/−^ double mutants at 4 dpf showed a pattern consistent with real time PCR result (Fig. 2E–H, N–Q).

### *klf2* mutants result in blunted Notch activation and reduced ventricle regeneration

Next we performed ventricle ablation in all three *klf2* homozygous mutants with *Tg(vmhc:mCherry-NTR)* background. Although *klf2a* was strongly upregulated in *klf2b*^−/−^ ablated hearts at 24 hpt and vice versa (Fig. 2I–L, R–U), the ventricular fluorescence and heart morphology of ablated hearts were not recovered in all three mutants at 4 dpt after injury compared to wildtype (Supplementary figure 2). We quantified the heart regeneration rate at 4 dpt and found the percentage of recovered larvae reduced from 73% in wildtype (N = 620) to 42% in *klf2a*^−/−^ mutants (N = 975) and 39% in *klf2b*^−/−^ mutants (N = 868). The percentage further reduced to 24% in *klf2*^−/−^ double mutants (N = 323) (Fig. 2W).

To examine whether *klf2a* and *klf2b* regulated ventricle regeneration through mediating Notch signaling activation, we bred the *klf2* mutants to homozygosity in *Tg(vmhc:mCherry-NTR; tp1:d2GFP)* background. In contrast to the strong activation of Notch signaling in the AVC of wildtype hearts after ventricle ablation, no such activation was observed in the three *klf2* mutants from 12 to 48 hpt (Fig. 2X–M’). WISH of *notch1b* expression also exhibited a similar pattern. *notch1b* expression was significantly upregulated in the AVC and expanded to chambers in wildtype ablated hearts. However, in both control and ablated *klf2* mutant hearts, *notch1b* was only expressed weakly in the AVC and OFT (Fig. 2N’–U’). These results indicated that both homologues of in human gene *KLF2* in zebrafish, *klf2a* and *klf2b*, were important molecules necessary for Notch signaling activation and cardiac regeneration.

### Endocardial primary cilia exist in the hearts at later stages

**Figure 3:**
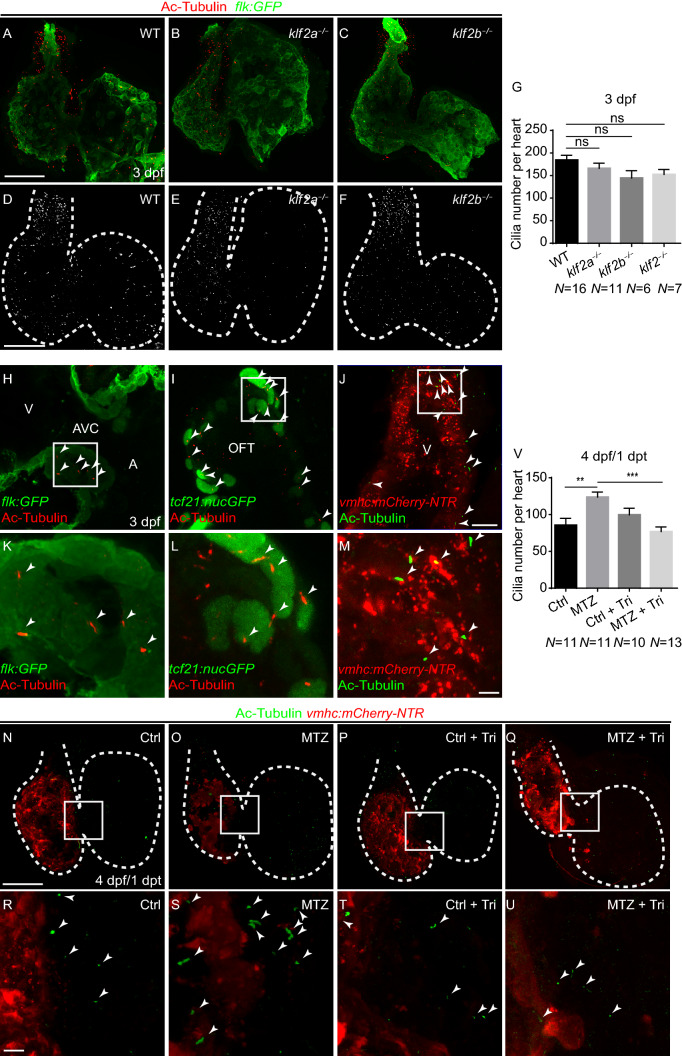
**Endocardial primary cilia exist in the hearts at later stages**. (A–F) Confocal stack projections of immunofluorescence showing cardiac cilia in wildtype and *klf2* mutants at 3 dpf. Green, anti-GFP immunostaining; red, acetylated tubulin immunostaining. (D–F) greyscale of red channel only. (G) Quantification of cardiac cilia number in wildtype and *klf2* mutants at 3 dpf (N = 16, 11, 6, 7 respectively). Mean + s.e.m. ANOVA analysis, ns, not significant. (H–M) Acetylated tubulin immunostaining showing primary cilia in endocardial cells (H, K), epicardial cells (I, L), and cardiomyocytes (J, M) at 3 dpf. (K–M) magnified white boxes in (H–J). (N–U) Confocal stack projections of immunofluorescence showing cardiac cilia in controlTricaine or BDM treatment or ablated *Tg(vmhc:mCherry-NTR)* hearts with or without Tricaine treatment at 1 dpt. (R–U) magnified white boxes in (N–Q). Red, mCherry fluorescence; green, acetylated tubulin immunostaining. (V) Quantification of cardiac cilia number in control or ablated hearts with or without Tricaine treatment at 1 dpt/4 dpf (N = 11, 11, 10, 13 respectively). Mean + s.e.m. ANOVA analysis, **P < 0.01, ***P < 0.001. Scale bars, (A–F, N–Q) 50 µm, (H–J) 10 µm, (K–M, R–U) 5 µm. dpf, days post fertilization, dpt, days post treatment. Dashed lines outline the hearts. Arrowheads point to cilia. A, atrium; AVC, atrioventricular canal; OFT, outflow tract; V, ventricle

Knowing that *klf2a* and *klf2b* could respond to hemodynamic alteration and regulate Notch signaling activation as well as ventricle regeneration, we then aimed to explore how blood flow change mediated *klf2* gene expression. Primary cilia, among others, are the popular candidate mechanical sensors for shear stress in endothelial cells in various models (Nauli et al., 2008). Previous study revealed the existence of primary cilia in zebrafish heart at 1 dpf (Samsa et al., 2015). However, whether primary cilia still exist at later stages when we perform ventricle ablation study is still unclear. Immunofluorescent staining of acetylated-alpha tubulin, a primary cilia marker, was performed at 3 dpf. Multiple signals could be detected at this stage, most in the OFT and AVC region as well as in the ventricle and atrium at lower abundance (Fig. 3A, D). To verify the location of these primary cilia, we performed acetylated-alpha tubulin immunostaining using *Tg*(*flk:GFP*), *Tg*(*tcf21:nucGFP*) and *Tg*(*vmhc:mCherry-NTR*) fishlines. The results indicated cilia existed in all three layers of the heart, including endocardial cells around AVC (Fig. 3H, K), epicardial cells in OFT (Fig. 3I, L) and ventricular CMs (Fig. 3J, M). We also examined the localization of Arl13b, a small GTPase of the Arf/Arl family that could bind to primary cilia, with immunostaining and reporter line *Tg(Ubi:Arl13b-GFP)* (Austin-Tse et al., 2013). Although most Arl13b signal was on the plasma membrane, primary cilia-like structure could be detected in the AVC region of 3 dpf zebrafish heart (Supplementary figure 3). Thus our results confirmed that cardiac primary cilia existed before ventricle ablation and there was some cilia located in the AVC endocardium where Notch signaling was activated.

We then examined the primary cilia formation in the *klf2* mutants with acetylated-alpha tubulin immunostaining (Fig. 3B, C, E–G). The result indicated the number of cilia had no significant change in the hearts of *klf2a*^−/−^ mutants (166.0 ± 11.7 per heart, N = 11), *klf2b*^−/−^ mutants (144.5 ± 16.5 per heart, N = 6) and *klf2*^−/−^ double mutants (152.4 ± 11.2 per heart, N = 7) compared to wildtype (184.2 ± 10.7 per heart, N = 16) at 3 dpf. We further characterized the primary cilia pattern in the injured *Tg(vmhc:mCherry-NTR)* larvae hearts (Fig. 3N–V). The cardiac cilia number in the control group at 4 dpf was dramatically reduced (85.5 ± 9.5 per heart, N = 11) compared to that of 3 dpf mentioned above (Fig. 3G, V). However, the staining in ablated hearts at 4 dpf/1 dpt revealed an increase in the cilia number (123.7 ± 7.0 per heart, N = 11) which could be obliterated by blood flow reduction induced by post-ablation Tricaine treatment (76.9 ± 6.3 per heart, N = 13). Our results suggested a dynamic pattern of primary cilia formation in the heart during development and regeneration, which was probably regulated by hemodynamic alteration.

### Cilia knockdown inhibits *klf2* and Notch activation during ventricle regeneration

**Figure 4:**
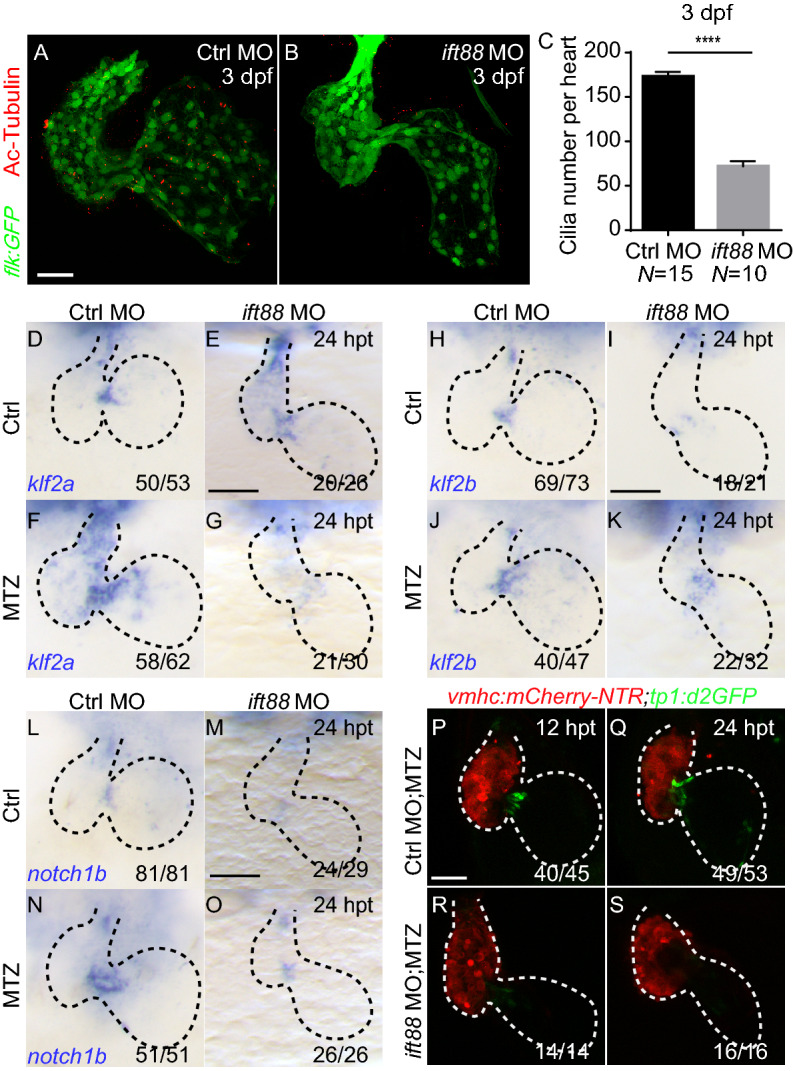
**Cilia knockdown inhibits Notch signaling and*klf2*activation during ventricle regeneration**. (A, B) Acetylated tubulin immunostaining in *Tg(flk:GFP)* heart showing cardiac cilia in control morphants (A) and *ift88* morphants (B) at 3 dpf. Green, anti-GFP immunostaining; red, acetylated tubulin immunostaining. dpf, days post fertilization. (C) Quantification of cardiac cilia number in control morphants and *ift88* morphants at 3 dpf (N = 15, 10 respectively). Mean + s.e.m. Student’s *t*-test, ****P < 0.0001. (D–G) Whole-mount *in situ* hybridizations indicated *klf2a* upregulation in control morphant ablated hearts (F) compared to control hearts (D) at 24 hpt, whereas this activation was blocked in ablated *ift88* morphant hearts (G). (H–K) Whole-mount *in situ* hybridizations indicated *klf2b* upregulation in control morphant ablated hearts (J) compared to control hearts (H) at 24 hpt, whereas this activation was blocked in ablated *ift88* morphant hearts (K). (L–O) Whole-mount *in situ* hybridizations indicated *notch1b* upregulation in control morphant ablated hearts (N) compared to control hearts (L) at 24 hpt, whereas this activation was blocked in ablated *ift88* morphant hearts (O). (P–S) Confocal stack projections of ablated *Tg(vmhc:mCherry-NTR; tp1:d2GFP)* hearts indicated Notch signaling activation was inhibited in *ift88* morphants at 12–24 hpt. Scale bars, 50 µm. hpt, hours post treatment. Dashed lines outline the hearts. Numbers indicate the ratio of representative staining observed

To further investigate the role of primary cilia in ventricle regeneration, we knocked down the expression of *ift88*, an important intraflagellar transporter involved in the development and maintenance of primary cilia (Pazour et al., 2000). After *ift88* morpholino injection at one cell stage, acetylated-alpha tubulin immunostaining showed the cilia formation was disrupted in the developing inner ear and pronephric duct, two well-known ciliated organs (Supplementary figure 4A, D, E, H). These phenotypes were similar as seen in *ift20*^−/−^*and ift172*^−/−^ mutants (Supplementary figure 4B, C, F, G) and assured the effectiveness and specificity of *ift88* knockdown. We then examined the primary cilia formation in the hearts of *ift20*^−/−^, *ift172*^−/−^ mutants and *ift88* morphants at 3 dpf. The number of cilia was dramatically reduced in *ift20*^−/−^, *ift172*^−/−^ mutants and *ift88* morphants (57.4 ± 8.9, 49.9 ± 5.4, 71.9 ± 6.0 per heart, N = 7, 7, 10, respectively) compared to control (173.1 ± 5.3 per heart, N = 15) (Figs. 4A–C, Supplementary figure 4I–M). These results confirmed that *ift88* knockdown disrupted cardiac primary cilia formation as in other *ift* family gene mutants.

We further performed ventricle ablation in *ift88* MO injected *Tg(vmhc:mCherry-NTR)* larvae to examine the activation of *klf2* expression and Notch signaling. Although *klf2a* expression was upregulated in the ventricle of *ift88* morphants without injury, the activation of *klf2a* and *klf2b* as seen in the ablated hearts of control morphants was inhibited in the ablated hearts of *ift88* morphants (Fig. 4D–K). Similarly, Notch signaling activation in injured hearts was also blocked in *ift88* morphants, revealed by WISH of *notch1b* mRNA and *Tg(tp1:d2GFP)* reporter line (Fig. 4L–S). To examine the effect of cilia knockdown on the expression of cardiac transcription factors after heart injury, we performed WISH of *hand2* and *nkx2.5*. The results showed both genes were highly enhanced in the ablated hearts of control morphants, whereas these activation were blunted in the ablated hearts of *ift88* morphants (Supplementary figure 5). Taken together, these findings confirmed the requirement of cardiac primary cilia for the activation of *klf2* and Notch signaling during ventricle regeneration.

## Discussion

**Figure 5:**
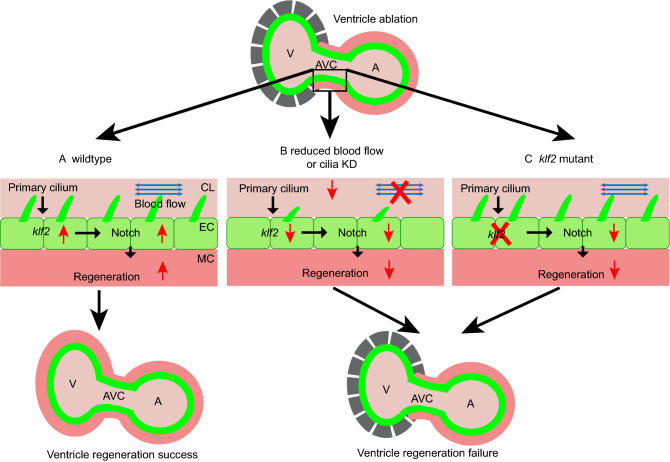
**Diagrams of hemodynamic-responsive Klf2-dependant Notch activation in ventricle regeneration**. (A) During normal ventricle regeneration process, primary cilia on the endocardial cells (green) sense the oscillatory blood flow (blue arrows), which leads to upregulation of the *klf2* and subsequent activation of Notch signaling in the endocardium. This Notch activation is essential for myocardium (red) regeneration. (B) When blocking blood flow or impairing cilia development, endocardial *klf2* expression and Notch signaling activation are inhibited, which lead to failure of ventricle regeneration. (C) In *klf2* mutants, the lack of endocardial *klf2* gene expression affects the activation of Notch signaling, and the damaged ventricle cannot regenerate. A, atrium; AVC, atrioventricular canal; CL, cardiac lumen; EC, endocardium; KD, knockdown; MC, myocardium; V, ventricle

In this study we have revealed the unique role of mechanosensation pathway in regulating cardiac regeneration. After ventricle ablation, hemodynamic alteration is perceived by endocardial cells through primary cilia, which mediates the upregulation of hemodynamic responsive factors *klf2a* and *klf2b.* The increased *klf2* gene expression in turn activates endocardial Notch signaling to promote ventricle regeneration (Fig. 5, left panel). When blood flow is significantly reduced by Tricaine or BDM treatment, or disruption of cardiac primary cilia formation occurs as in *ift88* morphants, there is no sufficient extrinsic mechanical signal to be sensed or transmitted to stimulate *klf2* upregulation which results in impairment of Notch signaling activation and failure of heart regeneration (Fig. 5, middle panel). If the *klf2* genes are knocked out, Notch signaling activation is also blocked and the heart will not be able to regenerate normally (Fig. 5, right panel).

### *klf2a* and *klf2b* both respond to hemodynamic alteration

The KLF proteins of Krüppel-like factor family are widely expressed transcriptional regulators which play important roles in hematopoiesis, angiogenesis, fin and epidermal development (Atkins and Jain, 2007). Specifically, KLF2 is one of the most popular blood flow responsive molecules (Dekker et al., 2002). Goddard et al. have revealed that hemodynamic forces affect heart valve development through the KLF2-WNT9B paracrine signaling axis in mice, and *klf2a*, one of the two *Klf2* homologue genes in zebrafish, performs a similar function (Goddard et al., 2017). *klf2a* can also regulate fibronectin synthesis by participating in mechanical conduction to affect zebrafish heart valve morphogenesis (Steed et al., 2016). However, *klf2b*, the other zebrafish homologue, obtains much less attention. Recently Rasouli et al. have reported that while single mutants of *klf2a*^−/−^ or *klf2b*^−/−^ exhibit no gross defects, *klf2*^−/−^ double mutants display a novel phenotype of CM extrusion towards the abluminal side which also requires cardiac contractility (Rasouli et al., 2018). However, we did not notice obvious CM extrusion in our double mutants, presumably due to various truncated products in different mutant alleles. Most of our *klf2*^−/−^ double mutant larvae cannot survive into adulthood which is consistent with previous report (Rasouli et al., 2018). In our study we have shown both *klf2a* and *klf2b* can respond to hemodynamic alteration after ventricle injury. Expression of both genes initially increases in the AVC region while only *klf2a* expands to the ventricle and atrium at later stages. Interestingly the expression of *klf2a* in *klf2b*^−/−^ mutants or *klf2b* in *klf2a*^−/−^ mutants is upregulated during heart regeneration, probably due to compensation for the loss of the other paralogue. However, both *klf2a* and *klf2b* are required for endocardial Notch activation since such activation is blocked in single mutants which results in regenerative failure. We speculate that Klf2a and Klf2b may form a complex to coordinately regulate Notch expression directly or indirectly, which requires further investigation in the future.

### Primary cilia act as a link between hemodynamics and Notch signaling

Primary cilia on endothelium are involved in fluid flow sensation which can transduce shear stress signal into functional responses (Nauli et al., 2008). Liu et al. reported that primary cilia regulate hematopoietic stem and progenitor cell specification through Notch signaling in zebrafish (Liu et al., 2019). Samsa et al. demonstrated that primary cilia are present in the embryonic zebrafish endocardium at 30 hpf, and shear stress promotes the development of cardiac trabeculae by promoting the expression of *notch1b* through primary cilia (Samsa et al., 2015). So we speculate that primary cilia may participate in the mechanical shear force sensation and regulation of Notch signaling during cardiac regeneration at later stages. In this study we first confirmed the presence of cardiac cilia at 3 and 4 dpf in the endocardium of AVC region where Notch signaling is activated during heart regeneration. Primary cilia can also be detected in the ventricle, atrium and OFT, where they are not confined to endothelial cells but in the CMs and epicardial cells. The physiological functions these primary cilia possess are still unclear at this moment. Primary cilia formation is sensitive to flow speed and patterns (Egorova et al., 2011), and we did observe a highly dynamic pattern in the number of cardiac cilia. The cilia number in 4 dpf heart is much less than that in 3 dpf in physiological situation, but it increases after ventricle ablation and this increase can be blunted by inhibition of blood flow. Espinha et al. also demonstrated an increase in microtubules around primary cilia in response to oscillatory fluid flow stimulation (Espinha et al., 2014). Next we knocked down *ift88*, an intraflagellar transporter essential for the assembly and maintenance of cilia (Pazour et al., 2000), to inhibit cilia formation. Although *ift88* morphant hearts still can contract and maintain low blood flow, the number of cilia is dramatically reduced. The expression of *klf2* genes is down-regulated and no Notch activation is observed in *ift88* morphant hearts after ventricle injury, indicating the importance of cilia in this regenerative process. Recently Villalobos et al. also reported the presence of primary cilia in adult mouse, rat and human hearts where they play a pivotal role in pathological cardiac remodeling after injury (Villalobos et al., 2019).

How primary cilia regulate *klf2* expression and affect Notch activation remains to be explored. Cilia are well known to participate in Hedgehog signaling during development (Rohatgi et al., 2007), they are also specialized calcium signal receptors that are highly sensitive to the low-frequency shear stress (Delling et al., 2013). There are a large number of ion channels on the primary cilia, like Piezo1/2, Polycystins 1/2, and other TRP channels (Orr et al., 2006). Piezo1 and piezo2 are important cation channels responsible for the mechanical activation in the somatosensory system (Coste et al., 2010). Polycystins 1/2 can sense fluid flow and transduce signaling on primary cilia of mouse embryonic kidney epithelial cells (Nauli et al., 2003). Polycystins 2 (also known as Trpp2) and Trpv4 ion channels play a regulatory role between blood flow and *klf2a* gene expression during heart valve formation (Heckel et al., 2015) and *trpv4*^−/−^ mutants impede heart regeneration (Gálvez-Santisteban et al., 2019). However, whether these ion channels exist on the primary cilia of endocardial cells and how they participate in the regulation of heart regeneration warrant further investigation.

In summary, this study demonstrates primary cilia and flow responsive Klf2a/Klf2b factors as the mechanistic link between hemodynamic alteration and Notch signaling activation. Our findings reveal the pivotal role of mechanosensation pathway in regulating heart regeneration and provide novel insights and new directions for the treatment of ischemic heart diseases.

## Methods

### Zebrafish husbandry

Zebrafish were raised and maintained under standard conditions. All experiments were performed according to institutional and national animal welfare guidelines. The zebrafish lines used in this study were as follows: *Tg(tp1:d2GFP)* (Clark et al., 2012), *Tg(vmhc:mCherry-NTR)* (Zhang et al., 2013), *Tg(Ubi:Arl13b-GFP)* (Austin-Tse et al., 2013), *Tg(flk:GFP)* (Jin et al., 2005), *Tg(tcf21:nucGFP)* (Mandal et al., 2017), *ift20*^−/−^ and *ift172*^−/−^ (courtesy of Dr Ying Cao). In all experiments, embryos and larvae over 24 hpf were maintained in E3 water with 0.003% PTU (1-phenyl-2-thiourea, Sigma, P7629) to prevent pigmentation.

### Generation of mutant zebrafish

*klf2a*^−/−^ mutants, *klf2b*^−/−^ mutants and *klf2a*^−/−^/*klf2b*^−/−^ double mutants were generated using CRISPR/Cas9 technique (Chang et al., 2013). The Cas9 mRNA was generated by *in vitro* transcription from a linearized plasmid pT3TS-nls-zCas9-nls (CZRC, China) using a mMESSAGE mMACHINE kit (Ambion). sgRNA target sites were identified using web-based tool ZiFiT (http://zifit.partners.org). The sgRNAs were *in vitro* transcribed from the DNA template of PCR-amplified products of the pMD19-T-gRNA vector (CZRC, China) with specific forward primers (*klf2a*: 5′-TGTAATACGACTCACTATAggtggaaggatgaactggacGTTTTAGAGCTAGAAATAGC-3′ and *klf2b*: 5′-TGTAATACGACTCACTATAggatgcaacccagcatgcagGTTTTAGAGCTAGAAATAGC-3′), and a universal reverse primer (5′-AAAAAAAGCACCGACTCGGTGCCACT-3′). A mixture of 300 pg Cas9 mRNA and 60 pg *klf2a* or *klf2b* sgRNA was injected into wildtype embryos at one-cell stage to obtain corresponding single mutants. Double mutants were generated by injecting Cas9 mRNA and *klf2b* sgRNA into homozygous *klf2a*^−/−^ mutant embryos. Positive founders were mated with wildtype fish to obtain F1 generation. The F1 heterozygous zebrafish with identical frameshift mutations were intercrossed to generate F2 homozygous mutants.

### Chemical treatment

*Tg(vmhc:mCherry-NTR)* larvae at 72 hpf were treated with 6 mmol/L MTZ (Metronidazole, Sigma) in E3 water for 4 h as previously described (Zhang et al., 2013). As controls, age-matched *Tg(vmhc:mCherry-NTR)* siblings were incubated in 0.2% DMSO (dimethyl sulfoxide, Fisher Scientific) in E3 water for the same period. To stop blood flow, control or ablated *Tg(vmhc:mCherry-NTR)* larvae were treated with 1.8 mmol/L Tricaine (3-aminobenzoic acid ethyl ester, Sigma) or 10 mmol/L BDM (2,3-Butanedione monoxime, Sigma) in E3 water from 15 hpt for 9 h at 28 °C. Treated larvae were washed three times with fresh E3 water at the end of ablation, Tricaine or BDM treatment and then allowed to continue to grow in fresh E3 water.

### *In situ* hybridization

Whole mount *in situ* hybridization was performed as previously described (Zhang et al., 2013), using the following probes: *notch1b*, *klf2a*, *hand2*, *nkx2.5*. The primers used for *klf2b* probe synthesis were: forward primer 5′-GAATTCCGCACACAATTGGTCTAGGA-3′ and reverse primer 5′-GGTAATACGACTCACTATAGGTACGTACATCGTTGTGCATTTTCCAC-3′.

### Immunofluorescence

Immunofluorescence staining on dissected larval hearts or whole mount larvae was performed as previously described (Zhang et al., 2013). The primary antibodies used in this study include: anti-GFP (chicken; Abcam, ab13970), anti-acetylated alpha tubulin (mouse; Abcam, ab24610), anti-Arl13b (rabbit; Proteintech, 17711-1-AP). The secondary antibodies used in this study include: Alexa Fluor 488 goat anti-mouse IgG, Alexa Fluor 488 goat anti-rabbit IgG, Alexa Fluor 555 goat anti-mouse IgG, Alexa Fluor 555 goat anti-rabbit IgG and Alexa Fluor 488 goat anti-chicken IgG from Invitrogen. Fluorescent images were obtained using a Leica SP8 or Zeiss LSM710 or Zeiss LSM880 confocal microscope.

### Morpholino injection

Morpholino injections were performed as previously described (Samsa et al., 2015). The morpholinos against *ift88* (5′-CTGGGACAAGATGCACATTCTCCAT-3′) was purchased from GeneTools. 5 ng *ift88* MO was injected into embryos at one-cell stage. Embryos and larvae at specific stages were used for relevant experiments.

### Quantitative real-time PCR

qPCR analysis for global expression of *klf2a* and *klf2b* was performed on cDNA obtained from 4 dpf wildtype, *klf2a*^−/−^ mutants, *klf2b*^−/−^ mutants and *klf2*^−/−^ double mutants. Total RNA was extracted by homogenizing 30 embryos in TRIzol reagent (Invitrogen) with a TGrinder pestle (Tiangen). 2 µg total RNA was used for cDNA synthesis with a ReverTra Ace qPCR RT Kit (TOYOBO). All real-time PCR reactions were performed in quadruplicate with PowerUp™ SYBR® Green Master Mix (Thermo Fisher) and three independent biological repeats were performed. Gene expression values were normalized using β-actin as internal control. For relative quantification analysis, statistical significance was determined by ANOVA analysis. The primers used in this study were as follows: *klf2a* forward primer 5′-GCTGGGAGAACAGGTGGAAGG-3′, reverse primer 5′-GCCATGCCGAGTCCGAGATT-3′; *klf2b* forward primer 5′-ATGCAGCGGGCTCTTCTCAC-3′, reverse primer 5′-TTTTCACCGGTGTGAGTGCG-3′.

### Quantification and statistical analysis

The regeneration ratio was calculated as the number of recovered larvae over the number of total injured larvae. Values were presented as mean ± s.e.m. Statistical significance was defined as a threshold of *P* < 0.05 determined by Student’s *t*-test between two groups, ANOVA analysis between more than two groups or Binomial test in quantification of the percentage of recovered hearts.

## Electronic supplementary material

Below is the link to the electronic supplementary material.

Supplementary material 1 (PDF 1092 kb)
