# Supplementary material for: Primary cilia mediate Klf2-dependant Notch activation in regenerating heart
Source: Protein Cell. 2020 Apr 5;11(6):433–45. doi: 10.1007/s13238-020-00695-w (PMC7251007; doi:10.1007/s13238-020-00695-w)
Supplement: Supplementary file 1 — Supplementary material 1 (PDF 1092 kb) [file 13238_2020_695_MOESM1_ESM.pdf]

Supplementary materials

Supplementary figure 1 Reduced blood flow attenuates Notch signaling activation and inhibits ventricle regeneration.

Supplementary figure 2 Reduced blood flow or *klf2* gene knockout inhibits ventricle regeneration.

Supplementary figure 3 Cilia exist in endocardium of atrioventricular canal.

Supplementary figure 4 *ift* family gene knockdown or knockout impairs cilia development.

Supplementary figure 5 Cilia knockdown affects expression of cardiac transcription factors after heart injury.

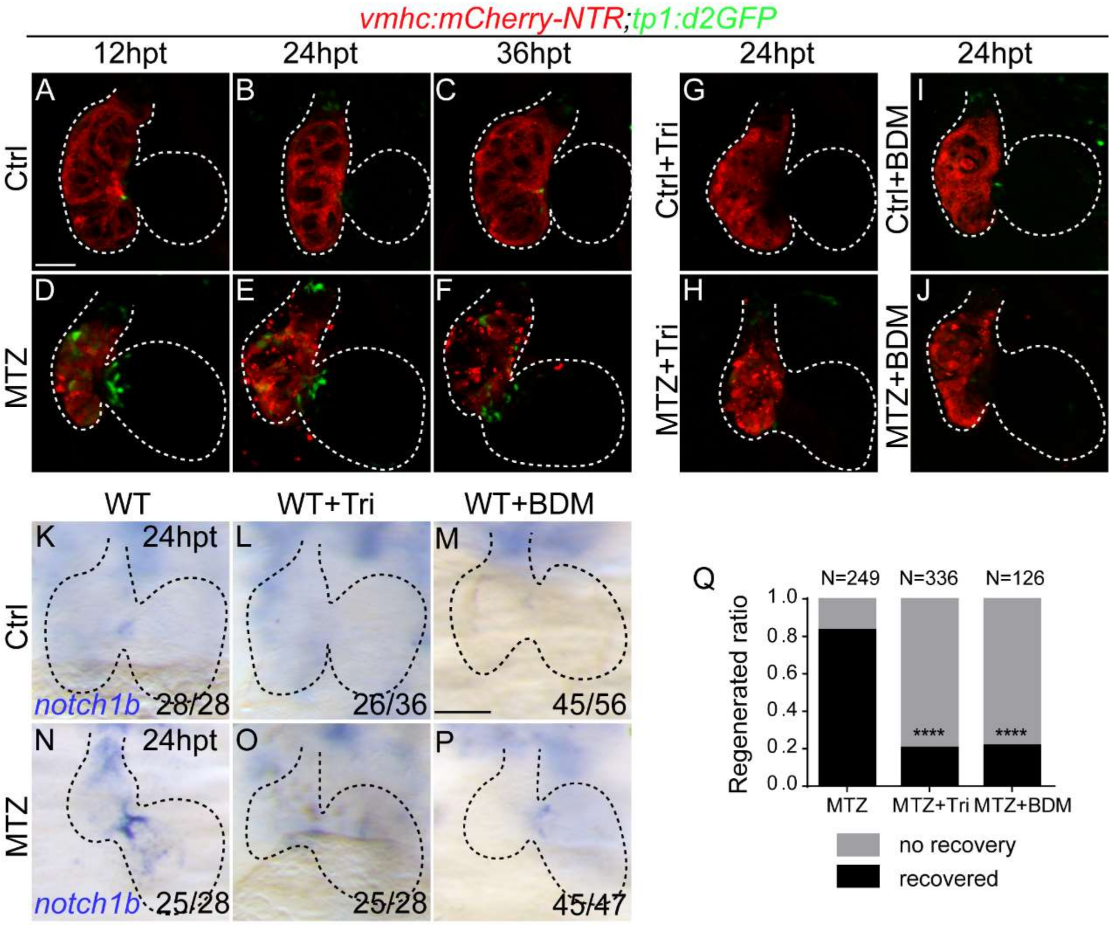

Supplementary figure 1. Reduced blood flow attenuates Notch signaling activation and inhibits ventricle regeneration.

(**A-J**) Confocal stack projections of *Tg(vmhc:mCherry-NTR; tp1:d2GFP)* hearts showing Notch signaling pattern in control (Ctrl) or ventricle ablated (MTZ) group without or with Tricaine and BDM treatment.

(**K-P**) Whole-mount *in situ* hybridizations indicated *notch1b* upregulation in ablated hearts (N) compared to control hearts (K) at 24 hpt, whereas this activation was blocked in Tricaine treated hearts (L, O) and BDM treated hearts (M, P). Numbers indicate the ratio of representative staining observed.

(**Q**) Quantification of the percentage of recovered hearts (black bars) of ablated group and Tricaine or BDM treated ablated group at 4 dpt. The number of larvae analyzed for each condition is indicated. Binomial test (versus MTZ), ****, $p<0.0001$.

Scale bars, 50 μm. hpt, hours post treatment. Dashed lines outline the hearts.

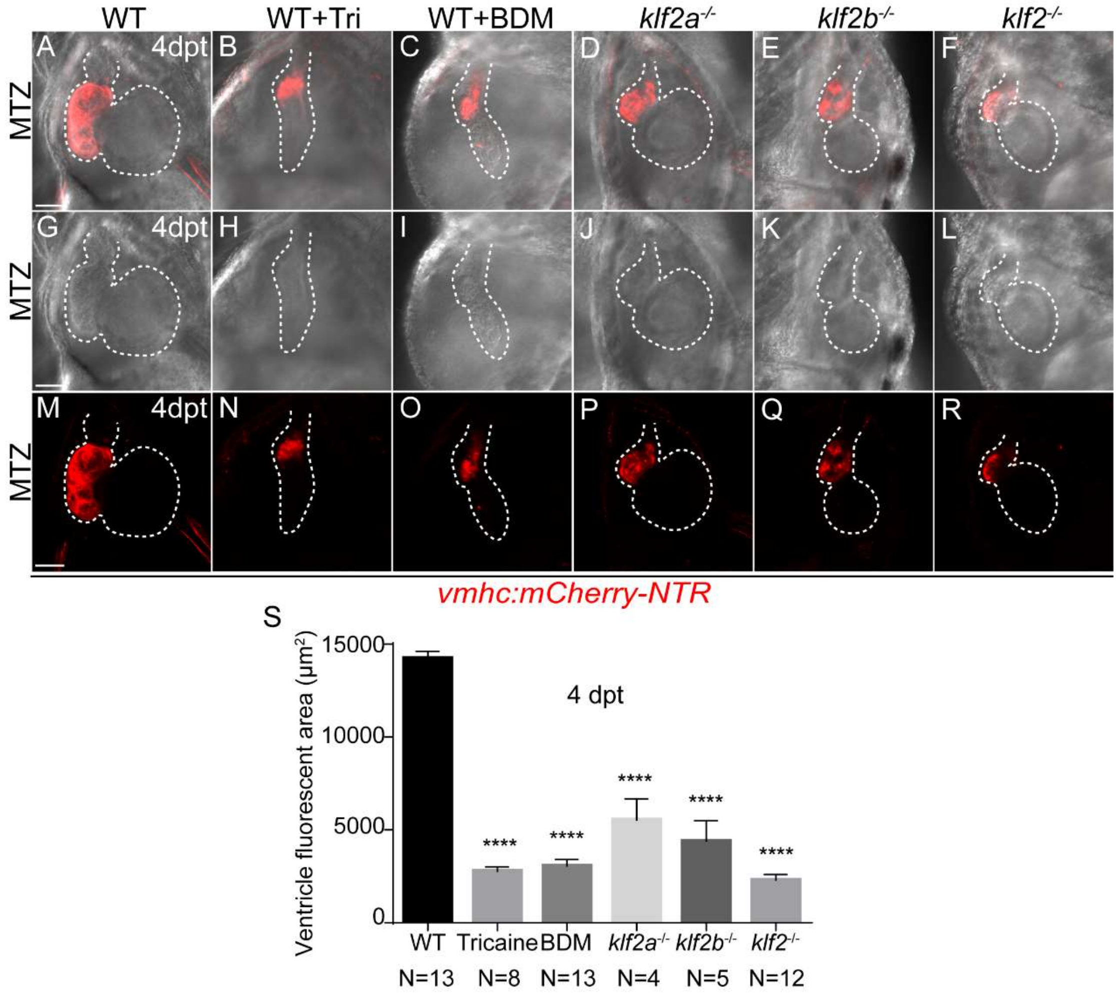

Supplementary figure 2. Reduced blood flow or *klf2* gene knockout inhibits ventricle regeneration.

(**A-R**) Confocal stack projections with bright field images showing the fluorescent pattern and morphology of ablated *Tg(vmhc:mCherry-NTR)* hearts, indicating near-total recovery in wildtype larvae (A, G, M), reduced or no recovery in Tricaine treated larvae (B, H, N), BDM treated larvae (C, I, O), *klf2a*$^{-/-}$ mutants (D, J, P), *klf2b*$^{-/-}$ mutants (E, K, Q) and *klf2*$^{-/-}$ double mutants (F, L, R) at 4 dpt. (G-L) bright field images only. (M-R) red fluorescence only. Scale bars, 50 μm. dpt, days post treatment. Dashed lines outline the hearts.

(**S**) Quantification of recovered ventricle area after injury at 4 dpt (N=13, 8, 13, 4, 5, 12 respectively). Mean + s.e.m. ANOVA analysis (versus WT), ****, p<0.0001.

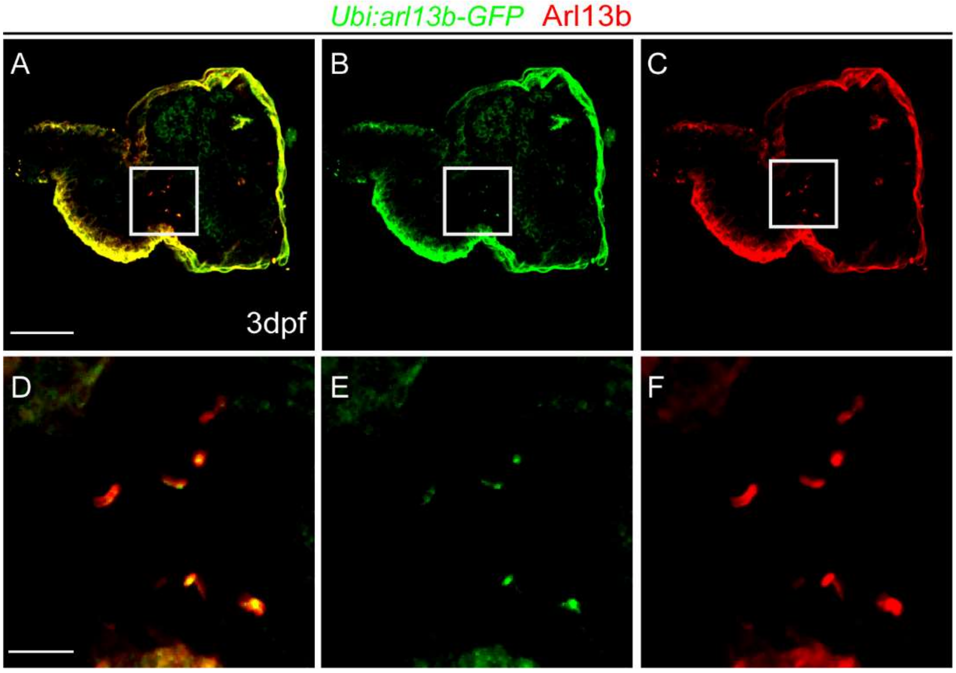

Supplementary figure 3. Cilia exist in endocardium of atrioventricular canal. (**A-F**) Arl13b immunostaining in *Tg(Ubi:arl13b-GFP)* heart showing endocardial cilia around atrioventricular canal at 3 dpf. (D-F) Magnified white boxes in (A-C). Green, anti-GFP immunostaining; red, Arl13b immunostaining.

Scale bars, (A-C) 50 µm, (D-F) 10 µm. dpf, days post fertilization.

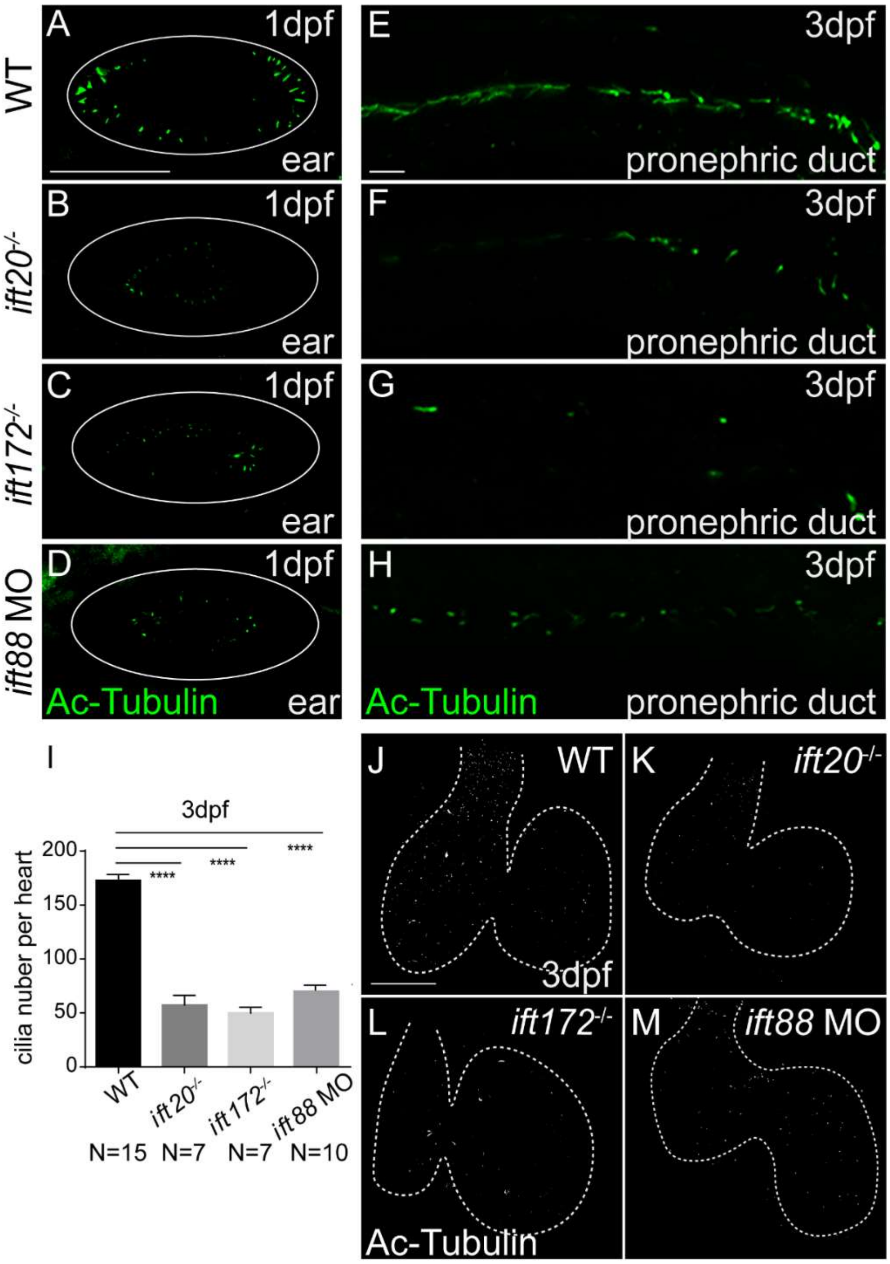

Supplementary figure 4. *ift* family gene knockdown or knockout impairs cilia development.

(**A-D**) Acetylated tubulin immunostaining indicated cilia development in ear (white oval) was impaired in *ift20*$^{-/-}$ mutants (B), *ift172*$^{-/-}$ mutants (C) and *ift88* morphants (D) compared to wildtype (A) at 1 dpf.

(**E-H**) Acetylated tubulin immunostaining indicated cilia development in pronephric duct was impaired in *ift20*$^{-/-}$ mutants (F), *ift172*$^{-/-}$ mutants (G) and *ift88* morphants (H) compared to wildtype (E) at 3 dpf.

(**I**) Quantification of cardiac cilia number in wildtype, *ift20*$^{-/-}$ mutants, *ift172*$^{-/-}$ mutants and *ift88* morphants at 3 dpf (N=15, 7, 7, 10 respectively). Mean + s.e.m. ANOVA analysis, ****, p<0.0001.

(**J-M**) Acetylated tubulin immunostaining in heart showing cardiac cilia in wildtype (J), *ift20*$^{-/-}$ mutants (K), *ift172*$^{-/-}$ mutants (L) and *ift88* morphants (M) at 3 dpf. White, acetylated tubulin immunostaining.

Scale bars, (A-D, J-M) 50 µm, (E-H) 10 µm. dpf, days post fertilization. Dashed lines outline the hearts.

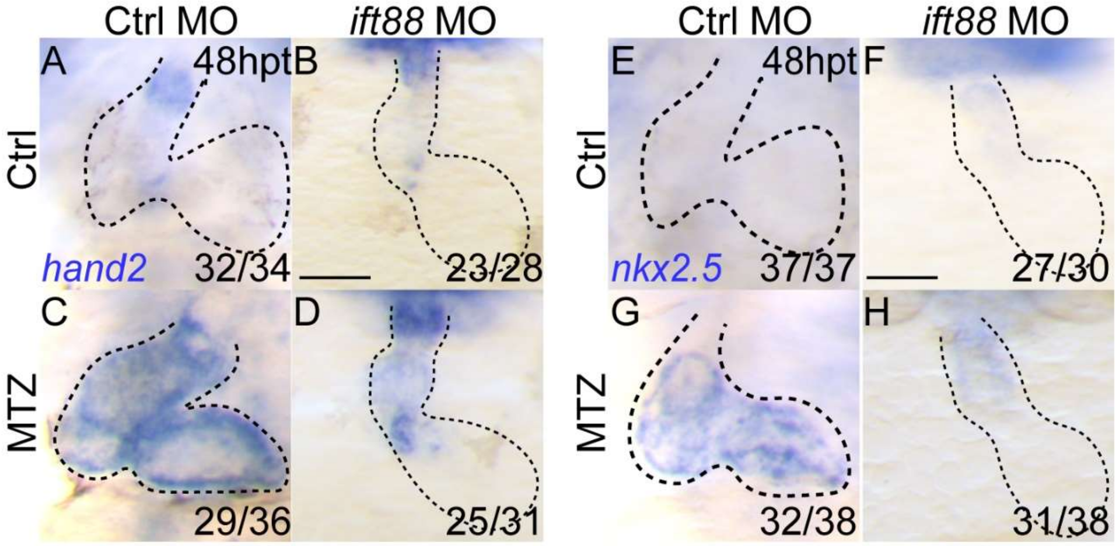

Supplementary figure 5. Cilia knockdown affects expression of cardiac transcription factors after heart injury.

(**A-D**) Whole-mount *in situ* hybridizations indicated *hand2* mRNA upregulation in control morphant ablated hearts (C) compared to control hearts (A) at 48 hpt, whereas this activation was blunted in *ift88* morphant ablated hearts (D).

(**E-H**) Whole-mount *in situ* hybridizations indicated *nkx2.5* mRNA upregulation in control morphant ablated hearts (G) compared to control hearts (E) at 48 hpt, whereas this activation was blunted in *ift88* morphant ablated hearts (H).

Scale bars, 50 μm. hpt, hours post treatment. Dashed lines outline the hearts. Numbers indicate the ratio of representative staining observed.
